# Digital interventions as part of routine addiction care in Sweden: healthcare staff perceptions of what works

**DOI:** 10.1186/s13722-025-00570-1

**Published:** 2025-05-12

**Authors:** Elisabeth Petersén, Hanna Augustsson Öfverström, Magnus Johansson, Christopher Sundström, Anne H. Berman

**Affiliations:** 1 Centre for Psychiatry Research, Department of Clinical Neuroscience, Karolinska Institutet, Stockholm, Sweden; 2 Stockholm Health Care Services, Region Stockholm, Stockholm, Sweden; 3 Procome Research Group, Medical Management Centre, Department of Learning, Informatics, Management and Ethics, Karolinska Institutet, Stockholm, Sweden; 4 Unit for Implementation and Evaluation, Center for Epidemiology and Community Medicine (CES), Stockholm, Sweden; 5 Department of Psychology, Uppsala University, Uppsala, Sweden

**Keywords:** Alcohol use disorder, Digital interventions, Addiction care, Healthcare staff, Thematic analysis

## Abstract

**Background:**

Every year about three million people die globally due to harmful alcohol use. The treatment gap remains high: only about 14% of individuals with problematic alcohol use access treatment. The implementation of digital interventions, addressing problematic alcohol use, into healthcare can be one way of reducing the treatment gap. However, little is known about how healthcare staff perceive the integration of digital interventions in routine addiction care. The aim of the study was to identify and describe healthcare staff’s experiences of perceived benefits of digital interventions for patients with alcohol use disorders (AUD), introduced in recent years within routine specialized addiction care in Sweden. The aim was further to explore how the use of such interventions in this setting could be further developed.

**Methods:**

This study was conducted as an exploratory qualitative interview study with 16 informants from addiction care staff in Stockholm, Sweden. The informants came from three different groups: clinical managers, staff referring outpatients to digital interventions from within addiction care, and therapists from an e-support unit. The interviews were recorded, transcribed, and analyzed with thematic analysis.

**Results:**

Three themes illustrated the benefits of digital interventions in routine addiction care, and future development areas. The theme *An easy way in* reflected the importance of easy access to addiction care where care would be flexible and available to everyone. *Meeting individual patient needs* raised the question of how to adapt treatment formats to the patients’ individual needs. *Smoothly interlocking organizational gears* highlighted the need for structure and cooperation between digital care and in-person care. Each theme included three additional subthemes.

**Conclusions:**

The study identifies key factors for successfully integrating digital interventions in addiction care, highlighting the importance of staff engagement, structured workflows, training, and ongoing evaluation using a sociological framework.

**Supplementary Information:**

The online version contains supplementary material available at 10.1186/s13722-025-00570-1.

## Background

According to the World Health Organization (WHO), three million deaths occur annually due to the harmful use of alcohol and approximately 230 different diseases in the world can be traced to alcohol consumption [1]. In addition, negative health consequences entail significant social and economic losses for both the individual and for society [2]. Despite the fact that there are currently several different types of treatment available for patients with Alcohol Use Disorder (AUD), a recent systematic review and meta-analysis demonstrates a large treatment gap, where up to 86% do not receive appropriate treatment for AUD [3]. Stigmatization of addiction-related diseases is one of the primary reasons why individuals with substance use disorders choose not to seek care [4]. For instance, a Danish study demonstrated that stigma reduces the likelihood that patients will seek healthcare for addiction-related issues [4]. This has sparked an increased discussion about how healthcare can reach patients who do not seek help and how treatment can be better tailored to individual needs [5]. Digital interventions show a large potential to reach more patients [6, 7]. For patients already in addiction treatment during the Covid-19 pandemic, digital interventions contributed to the maintenance of contact between healthcare staff and patients [8]. Digital interventions have been shown to reduce alcohol consumption for up to six months [9]; although a systematic review showed that longer follow-ups are lacking [10], recent findings suggest long-term benefits of both low- and high-intensity internet-based treatment programs for AUD [11].

Despite the potential of digital interventions, their integration into routine healthcare presents significant challenges. A Swedish study of psychiatric clinics showed that procedures for the assessment and treatment of substance use disorders was not fully aligned with national guidelines, and that local routines for such assessment and brief intervention were lacking to a large degree. Staff reported that a lack of knowledge and skills was one of the reasons, along with a need for further education and training [12]. Psychiatric healthcare professionals perceived that digital interventions for substance use screening and intervention could provide significant benefits for patients but indicated that they required training both in how to implement digital interventions as well as clear information about intervention contents [13]. Similary, a study evaluating a mobile application for problematic alcohol use, introduced at clinician visits in primary care, revealed that both patients and all healthcare professionals required clear implementation routines and guidelines to optimize its use [14].

Although there is a generally positive attitude toward digital interventions, healthcare professionals express concerns regarding changes to workflow [14] and increased workload [13]. Given that evidence-based digital interventions are still relatively new, there is a knowledge gap concerning how they can be effectively integrated into daily care. Therefore, it is critical that healthcare providers and policymakers develop strategies to facilitate the successful implementation of these interventions [7]. Implementation strategies should be informed by appropriate theoretical frameworks, as emphasized by Ross et al. [15], who highlight the importance of applying established theories to guide implementation processes.

Normalization Process Theory (NPT) [16] describes how new interventions are integrated into clinical practice and emphasizes the importance of adapting the implementation process to the competencies, workflows and organizational conditions of healthcare staff [17]. According to NPT, successful implementation is more likely when digital interventions align with health care staff´s competencies, goals and established work routines [15]. Moreover, understanding the specific context in which intervention is introduced, including the needs and capacities of the staff involved, is essential for ensuring its successful adoption [17]. A crucial aspect is collecting and analyzing feedback from both healthcare professionals and patients to identify barriers and facilitators for implementation [15, 17].

Previous research has explored the implementation of digital interventions in primary care [14, 18–20], yet limited knowledge exists regarding their integration into specialized addiction care. Implementation frameworks suggest that understanding the specific context of the treatment is crucial for identifying necessary adaptions in clinical routines and workflows [15]. Unlike other areas of healthcare, addiction care presents unique challenges, including high levels of stigma, complex patient needs, and varying levels of engagement with treatment [13]. For example, Alcohol use disorder (AUD) treatment differs from general healthcare as it encompasses a broad spectrum of medical and social interventions. The shared responsibility for addiction care (between healthcare services and social care in Sweden) necessitates specialized studies to understand and enhance cooperation between these sectors, ensuring more effective and integrated care for individuals with AUD [21, 22]. Given these complexities, understanding how digital interventions can be effectively embedded within addiction care is particularly important. Therefore, studies specifically dedicated to the implementation of digital interventions within addiction care settings are necessary to ensure that these interventions are appropriately adapted and effectively integrated into clinical practice.

The present study aimed to contribute new findings to reduce this knowledge gap by examining the implementation of digital interventions at the e-Support Unit (ESU) at the Stockholm Center for Dependency Disorders (SCDD). Established in 2018, the ESU provides digital interventions to patients who self-refer or are referred from outpatient units within the clinic as well as from external caregivers. The ESU provides digital interventions for a range of conditions, including alcohol, cannabis, tobacco, gambling, sleep disorders, as well as family support programs. The ESU employs a research-based approach, ensuring that interventions are continuously evaluated and refined [23]. Since its inception, over 6,000 individuals have initiated treatment programs, and the unit has experienced a threefold increase in staff, including nurses, therapists and physicians [24]. The ESU delivers online psychological treatment through module-based programs that include self-completed exercises based on cognitive behavior therapy and relapse prevention. Support is provided by therapists’ who primarily communicate with patients via secure written messages. Although video calls can be used at intake and telephone conversations can be added during treatment as needed, neither of these modalities features regularly in patient-provider contact. Patient access to treatment, provided on a secure online treatment platform available nationally in Sweden [25], requires electronic identification. Additionally, the ESU offers a public health intervention model, for individuals who prefer to remain anonymous, accessing treatment on a non-clinical platform.

This study specifically sought to explore the factors that have facilitated the successful implementation of digital intervention at the ESU. Our focus in this study is on fully digitalized interventions that are not integrated with face-to-face treatment, as seen in blended care, where research publications have been increasing (e.g [26, 27]).,. Rather than solely identifying barriers to implementation– a predominant focus in previous research [12, 28]– this study examined facilitators that have contributed to successful integration. By analyzing a setting where digital interventions have already been implemented, this study provides valuable insights into the mechanisms that support effective adoption and sustainability.

The specific objectives were (a) to identify and describe the perceived benefits of digital interventions currently available to outpatients with AUD within specialized addiction care, as reported by healthcare staff; and (b) to explore strategies for further enhancing the use and sustainability of such interventions within this setting. Informed by NPT, the study investigated how digital interventions have become embedded in addiction care routines and how they align with healthcare staff´s practices. By studying an established digital intervention program, the research aims to generate knowledge that can inform broader implementation efforts within addiction care, addressing critical gaps in the literature and guiding future efforts in similar health contexts.

## Methods

### Design and setting of the study

The study was conducted as an exploratory qualitative interview study. In Sweden, addiction care is structured for shared leadership between municipal social services and regionally managed healthcare. The medical aspects of addiction care are managed by specialized units in the regions. Respondents were staff recruited from clinical settings within the SCDD, a county-wide specialized addiction care clinic in Stockholm that provides inpatient emergency and rehabilitative care, outpatient care at multiple local clinics, as well as remote outpatient care via the ESU. Prior to initiating the study, this research and development project was anchored with the management group at the SCDD.

To recruit respondents, insight was needed regarding referral patterns to the ESU. Referrals can be sent from within the SCDD as well as from all healthcare providers (e.g., primary care, psychiatry and somatic treatment units) within the Stockholm region and, as needed, from providers in the rest of Sweden. Referrals both from within the SCDD and from outside healthcare providers are all sent directly to the ESU from the patient’s electronic healthcare record. To identify which specific units most frequently referred patients, we consulted the ESU for a list of the units they commonly receive referrals from. Based on this list, relevant units were contacted. Thereafter, interviewees were recruited from three different groups to cover different aspects of the research question: (1) ESU staff providing digital interventions, (2) healthcare staff working at SCDD who had been referring patients to the ESU, and (3) clinical managers at SCDD outpatient units as well as higher management levels.

### Participants and data collection

Recruitment for the study was based on purposive sampling [29]. Potential study participants from the three groups at the SCDD were contacted via email with a request for an interview as well as information about the purpose of the study. To target study participants with experience of referring patients to digital intervention, the ESU provided a list of clinical units that refer patients to the ESU’s digital interventions. One reminder was sent out to potential study participants. A total of 22 prospective study participants were contacted, of which 16 gave their consent to participate. The main reason for declining participation was lack of time. All interviews were conducted online via Teams, hosted by the Stockholm Region healthcare services and took place between November, 2022 and June, 2023. Of the 16 informants interviewed, eight were staff who referred patients for digital interventions delivered by ESU, four were healthcare staff from the ESU and four were clinical managers, working within the SCDD (see Table 1 for participant characteristics). The interviews lasted between 8 and 38 min, with an average duration of 21.5 min. All respondents were interviewed by EP, then a PhD candidate and trained specialist nurse in psychiatry with over 18 years of experience working in addiction care, and earlier experience of conducting both individual and focus group interviews. All interviews were based on an interview guide, see Additional File 1. All interviews were audio recorded and transcribed verbatim. The informants did not read the transcripts or the manuscript before publication.

**Table 1:** Participant characteristics

Participants (*n* = 16)						
Occupation	Participants		Type of informant			Mean age
	Female	Male	Referring participant	Manager	e-Support unit	
Physician	3	2	4	1	-	50
Psychologist	3	2	1	-	4	41
Nurse/specialist nurse	4	-	3	1	-	48
Other occupation	1	1	-	2	-	43
Total (*n* = 16)	11	5	8	4	4	46
Standard deviation (*n* = 16)						7.48

To maintain anonymity, managers with professions other than doctor, psychologist, or nurse were not specified.

### Data analysis

**Fig. 1:**
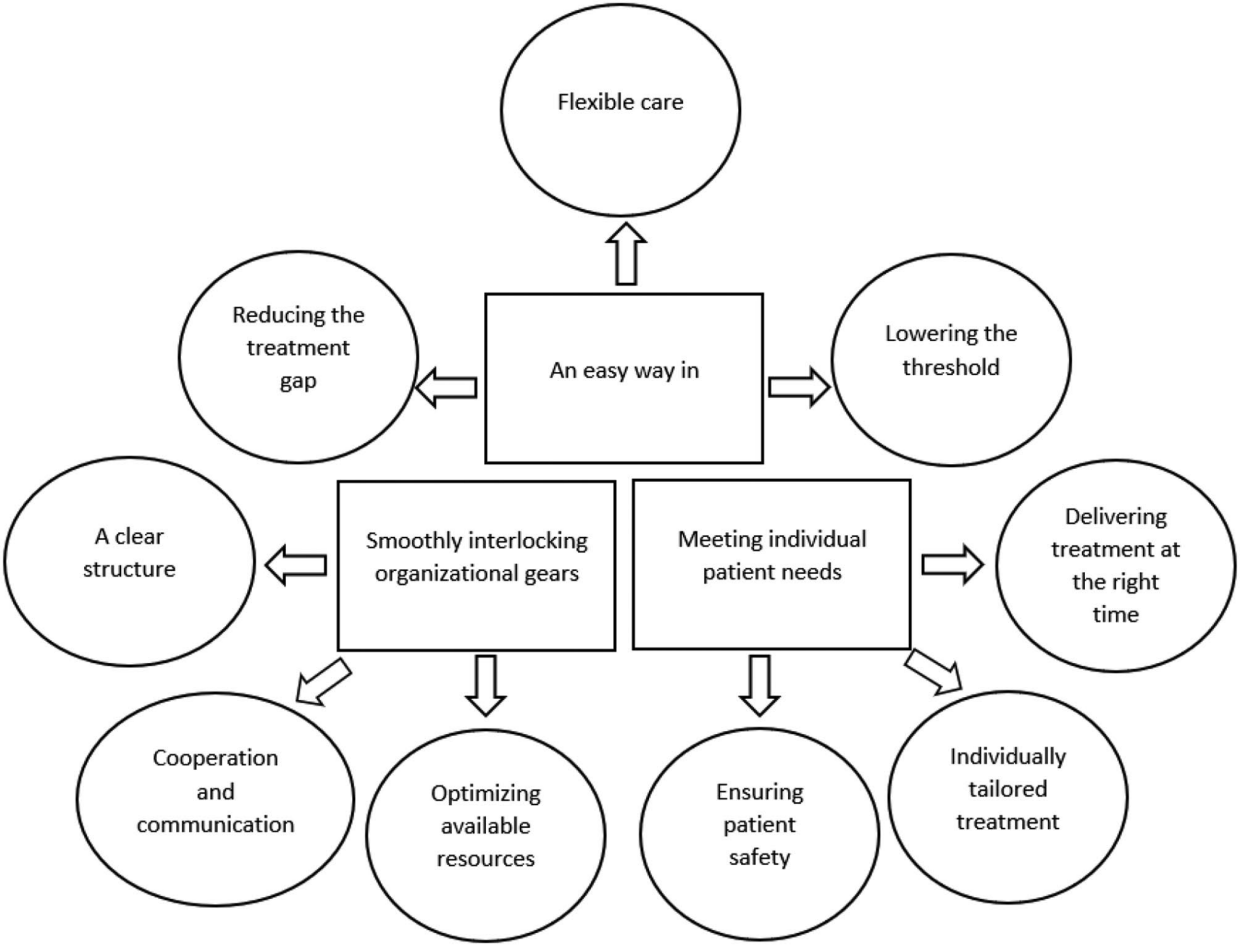
Final thematic map, showing the three themes with following sub-themes

Thematic analysis in six phases, as defined by Braun and Clarke [30, 31] was used to analyze the interviews, all manually transcribed by author EP. An inductive analysis was carried out; i.e., the data were analyzed without any predetermined themes or labels. In the first phase the material was transcribed, which also gave an opportunity to deepen familiarity with the material as it was listened to repeatedly during the transcription. When the material had been transcribed, it was read and reread several times and initial thoughts were noted [30]. Thereafter, to strive towards reliability and consistency in the analysis, consensus dialogues were initiated. In the first phase of this procedure, all transcribed interviews were de-identified and marked with a numerical ID, after which they were distributed between the authors for close reading and analysis. In the second phase all transcribed interviews were read through several times by the first author, and relevant phrases capturing the essence of the content were marked out. These phrases were then systematically compiled into groups with similar data, which in turn generated initial codes. In the third phase, the various codes were sorted into potential themes and in phase four, the emerging themes were reviewed by checking that they related to the extracted sentences and to the data set as a whole [30]. A total of three author meetings were held to discuss potential themes, sub-themes and codes during the process. This generated a basis for creating a thematic map of the analysis, on which the authors agreed (see Fig. 1 in the Results section and Table 2 under Author contributions for distribution of the analysis material). In the fifth phase, the emerging themes were defined, named and described in the methodological narrative. In the sixth phase, citations were selected and the results were compiled [30].

**Table 2:** Author distribution of completed analysis

Interview:	E. Petersén	M. Johansson	C. Sundström	A.H. Berman
No.1	X		X	
No.2	X	X		
No.3	X		X	
No.4	X			X
No. 5	X		X	
No.6	X	X		
No.7	X			X
No.8	X			X
No.9	X		X	
No.10	X		X	
No.11	X	X		
No.12	X	X		
No.13	X			X
No.14	X		X	
No.15	X	X		
No.16	X	X		

In the present study, we have chosen to discuss the results based on Normalization Process Theory (NPT). Normalization Process Theory is a framework that explains how new practices, such as innovations in healthcare or organizational procedures, become embedded in everyday routines. It focuses on the social processes that influence the implementation, integration, and sustainability of these practices. NPT identifies four key mechanisms: *coherence* (understanding the purpose and value of the practice), *cognitive participation* (commitment and engagement from individuals), *collective action* (the actual work required to implement the practice), and *reflexive monitoring* (ongoing evaluation and adaptation). By analyzing these factors, NPT helps researchers and practitioners understand why some innovations succeed while others fail [32]. The NPT framework is essential for understanding how complex interventions are diffused and adopted in organizations, emphasizing the collective and individual efforts required to achieve common goals. It focuses on the observable actions of implementing interventions and provides valuable insights for intervention design and evaluation processes. NPT serves as a sociological toolkit for analyzing the human processes involved in adopting new practices in different contexts, grounded in empirical studies and applicable in real-world settings [16].

## Ethics

Participants in the study were given information about the purpose and voluntary nature of their participation. They were assured that they could withdraw at any point without consequence. Verbal consent was obtained during the interview, with written consent following. No formal ethical approval was required according to Swedish Law (SFS 2003:460), as the study did not involve questions about personal health [33]. A previous similar study had received a consultative statement indicating no ethical approval was needed [13], and for the current study, such a consultative statement was provided by KI Ethics. Their decision was based on regulations regarding ethical review of research involving humans [33].

## Results

The analyses rendered three themes: (1) An easy way in, (2) Meeting individual patient needs, and (3) Smoothly interlocking organizational gears. The three themes in turn had three sub-themes each: Theme 1 sub-themes Reducing the treatment gap, Flexible care and Lowering the threshold; Theme 2 sub-themes Delivering treatment at the right time, Individually tailored treatment and Ensuring patient safety; and Theme 3 sub-themes A clear structure, Cooperation and communication and Optimizing available resources (see Fig. 1). The themes and sub-themes are described in detail below with illustrative citations.

### Theme 1: An easy way in

Overall, this theme reflects the finding that the informants highlighted the importance of easy access to addiction care. Apart from a consensus that addiction care should be available to everyone who needs it, it was viewed as important that the care be flexible to make sure that as many people as possible gain access to the care they need. Increasing access to care creates an opportunity to reduce the waiting time for treatment and thus maintains the patient’s motivation to change.

#### Sub-theme 1a: reducing the treatment gap

The informants thought that offering evidence-based digital interventions for addiction care can increase access to treatment and reduce stigma. While stigma can discourage patients from seeking help, digital interventions enable patients to seek care in a more private setting without others knowing. The digital alternative can thus help bridge the treatment gap for those with substance use disorders, providing more personalized care. The cost-effective and scalable nature of digital interventions also enables a larger proportion of patients, even those with co-morbidities, to receive treatment. Scaling up the digital approach could serve a larger population.

*Without this feeling of being extremely stigmatized*,* where I think that the ESU has its great merit… we can provide care which*,* in the long run*,* will hopefully be at a fairly cheap level. Where we can have large… groups which we can treat relatively quickly and thus provide interventions based on the degree of need. (Informant 2*,* manager)*

Several informants expressed a common goal of reaching out to more patients who are not receiving the care they need, in order to reduce the treatment gap. They envisioned providing internet-based support to patients who want it, expanding access to treatment beyond their own care organization. Staff emphasized the importance of sharing knowledge, evaluating current practices, and innovating new ways to reach and help more patients. The focus was on increasing the number of patients they can serve and making sure everyone has access to the care they need.

*I think we should reach more patients and I think the overall goal is for more patients to have access to internet-based support… it does not have to mean that all patients should go to us [ESU]… the important thing is that people have access to this type of treatment… But*,* I think we should have more patients*,* I think we should share our knowledge with others so*,* we should continue to evaluate what we do and I think we should invent new things. (Informant 5*,* ESU staff)*

#### Sub-theme 1b: flexible care

Flexible care meant, among other things, that it is possible for patients to receive treatment around the clock. Patients can thus adapt the treatment and complete treatment tasks to the times that suit them best. If the patient wants to work on the tasks early in the morning, late in the evening or in the middle of the night, it does not affect the treatment. It is possible to combine the treatment with work and holidays, as patients do not have to be at a special clinic.

*At the same time*,* it is a little more flexible*,* you may not need to fit times in the same way there*,* but you can go in when it works for you*,* and that is an advantage. (Informant 3*,* referring staff)*

Flexible care also meant an opportunity for blended care, where patients can combine traditional face-to-face contact with digital treatment to address specific aspects of their needs. Informants perceived that blended care offers flexibility and can complement routine care, providing hope for better methods to reach patients. Several informants highlighted the importance of developing blended treatment methods for clinics, with one informant stating that it is the next step towards the future. The blended approach is seen as a good opportunity and as a complement to existing treatments, especially for patients who prefer remote therapy or specific types of therapy like CBT.

*No*,* but I think that they [ESU] will continue to develop their work and their treatment offerings. And what I think they are doing well now is that they contribute to the work to develop blended working methods for the clinic*,* because I think that is the next step.(Informant 7*,* manager)*

#### Sub-theme 1c: Lowering the threshold

The informants highlighted that suffering from a substance use disorder and seeking help in addiction care can be perceived as difficult. To reduce the obstacles that the patient may experience and make it easier for patients to seek care, informants judged it important for patients to easily be able to get in touch with care providers. Help should be available quickly, without long waiting times, and the informants highlighted that it is important that the patient always feels welcome when they seek help. Having a low entry threshold can be interpreted as offering several different ways to establish contact with addiction care.

*You should always be greeted with a welcome*,* [and feel that] you’ve come to the right place…and I think that the ESU is a good way. [We can]… think of it as a very*,* very broad funnel into treatment. (Informant 2*,* manager)*

The informants also expressed that seeking help in addiction care can often feel difficult, as there is still a stigma around substance use disorders. Motivation is a feeling that can be present in the moment and therefore, the informants expressed that it is important to be able to offer help to the patients when they want it, to maintain the motivation that exists. Some of the informants expressed that contact with addiction care could occur in different ways and that it does not always have to involve a doctor’s appointment at a clinic. In some cases, it may be more important to prioritize quick contact with a healthcare provider rather than a scheduled visit to the clinic.

*I think that there should be an easy path into addiction care. Seeking addiction treatment is still… surrounded by stigma and then I think that it is important to make the step into care as easy as possible and maybe it can be via a chat function. (Informant 2*,* manager)*

By offering digital interventions to the patients, the informants felt that establishing a treatment contact was accelerated and that it contributed to patients being able to begin evidence-based treatment earlier. The possibility of seeking care yourself via a digital platform was perceived as very positive. At the same time, informants perceived the possibility for other units within the SCDD to refer to treatment via the internet both as a support but also as a quick contact route. With a high influx of patients and limited capacity at regular outpatient clinics, internet support was seen as essential. Extended waiting times were a concern, as motivation could wane before the date arrived for a clinic appointment.

*We have an incredibly large influx of patients*,* so I don’t think our clinic really has enough capacity to take these patients and then it’s fantastic that they can get this support via the internet. While they are having contact with our clinic. (Informant 4*,* referring staff)*

### Theme 2: Meeting individual patient needs

This theme concerned the informants’ visions of another type of flexibility that characterizes digital interventions, namely adaptations of treatment format and content to individual patients and their idiosyncratic needs. This theme thus illuminated a more complex facet of adaptivity, beyond the actual access to treatment, which was captured in the first theme.

#### Sub-theme 2a: delivering treatment at the right time

This sub-theme illustrates the informants’ perceptions of the capacity of addiction care to adapt specific treatment to the patient’s needs. Examining whether the patient has a functional ability that is suitable for digital treatment in cases of co-morbidity or, alternatively, finding a strategy to adapt the treatment to the patient’s specific functional ability, would contribute to better patient-centered care. This sub-theme also includes the informants’ perceptions of possibilities for enabling multiple alternatives for patient treatment. The informants expressed that offering digital interventions opens an opportunity to offer treatment on several different levels.

*But…as healthcare providers in public activities*,* we have a responsibility to take care of the most seriously ill and give them the resources they need. But we also have a responsibility towards the population*,* and to make this work*,* we need to get better at being efficient in the so-called healthier group. (Informant 7*,* manager)*

The informants saw digital interventions as introducing multiple ways to receiving care and multiple ways for exiting care. Sometimes a patient may need to have their initial contact with internet-based treatment, while during the treatment a need may arise for in-person support; in the latter case, the informants expressed that there should be an opportunity to move the care to a physical clinic or, conversely, to transfer treatment to the digital context. For example, this could occur through digital follow-up of patients who are about to end their care contact.

*So opioid-dependent patients*,* perhaps patients treated with Methylphenidate*,* patients with co-morbidities and then perhaps also patients with a major social problem*,* I think they can primarily count on us to see them in person at outpatient care. Then I think that in the next stage they might be able to go further in care and treatment to some maintenance phase*,* that instead of ending they could continue contact with the ESU and have motivational talks in the form of a booster session. So… to end the in-person care but continue digitally…. You could have an outpatient care exit that is from in-person outpatient care to digital outpatient care*,* follow-up and then closure. (Informant 2*,* manager)*

#### Sub-theme 2b: individually tailored treatment

This sub-theme highlights informants’ perceptions that adapting treatment to individual needs benefits not only the patient but also healthcare staff and other patients. By tailoring care appropriately, staff workload can be reduced, resources allocated more efficiently, and queue times shortened. While personalization of care is essential, treatment should always be designed in consultation with the patient, respecting their autonomy while aligning with scientific evidence and clinical practice. Informants described this as a balancing act—healthcare professionals must consider patient preferences while working within medical, ethical, and financial constraints. A key concern was ensuring that patients receive the appropriate level of care without unnecessary interventions or excessive costs. In some cases, alternative care formats, such as video consultations, were seen as enhancing accessibility and efficiency.

*If you have fairly small needs*,* we should not burden the patient with an extreme number of interventions that are hugely expensive because it usually does not work*,* but we should provide care at the level that is needed…* (Informant 2, manager)

In striving to enhance the individual precision of treatment provided to patients, informants identified a potential to reduce the risk of dropout due to dissatisfaction with care. They emphasized that poor adherence to treatment is a common challenge, often leading to deteriorating health and increased suffering for the individual. To address this issue, informants highlighted the importance of fostering a collaborative relationship between patients and healthcare professionals, which could strengthen patients’ sense of control, self-esteem, and overall confidence in engaging with treatment. Additionally, increasing the adaptability of treatment was seen as a key strategy to improve adherence and enhance the likelihood of long-term positive outcomes. Informants stressed that expanding and refining precision treatment requires further research on how to match the “right” intervention with the “right” patient. A more evidence-based approach to individualizing treatment could not only improve the effectiveness of interventions but also optimize resource allocation within healthcare systems.

*We have tried the treatments and evaluated some. But the patient group is kind of heterogeneous… you would like to have more knowledge about exactly which patients [the treatment] is suitable for. Then maybe you can sort of sharpen the inclusion criteria or sort of the assessment procedure so that you get it more right in some way… [so you more easily identify which patients need which treatments] I think.* (Informant 5, ESU staff)

#### Sub-theme 2c: ensuring patient safety

The informants expressed that it was very important to find a way to match the right treatment to the patients so that it is not unsafe for the patient, for example for patients with pronounced suicidal thoughts or suicide plans, or for patients suffering from a psychotic illness. It was important to ensure that patients do not get hurt, or end up without help– neither from addiction care nor psychiatry. In addition, the informants expressed that it was necessary for ´ patients to understand the care provided, so it proceeds as planned, and to identify and address any issues that arise. Informed and empowered patients who actively participate in their care can thus improve the overall safety and effectiveness of their treatment.

*There can be disadvantages when they are very unstable and very turbulent mentally… because [a crisis] can happen very quickly. Then I think that the most important thing is that the outpatient clinic takes its responsibility*,* so that it is stable and ready to receive patients when*,* for example*,* the emergency support signals that ‘oops*,* now we have received a signal from the patient that a deterioration has occurred*,* and the patient is feeling worse. Can you ask the patient to come in*,* because you have the in-person opportunity to follow up? (Informant 11*,* referring staff)*

The informants highlighted the importance of effective communication and cooperation between the ESU and the patient’s “home” outpatient clinic to enhance patient safety and prevent negative events. They expressed that effective teamwork involves all team members working towards a common goal, building on structured communication, and establishing mutual trust. It was important that the team members felt comfortable speaking up if they noticed any safety risks for the patient; for example, challenges arising with suicidal patients. Cooperation among healthcare teams with diverse expertise was perceived as crucial for providing patient-centered and safe healthcare.

*The only thing we have come across is that there has been a bit of a problem with suicidal patients if there is an elevated suicidal risk*,* which is often the case among our patients*,* and then there has been a bit of doubt about e-support*,* [that is] if the patient can cope with e-support. (Informant 4*,* referring staff)*

A new way of working could also raise concerns about the patient’s well-being, where the healthcare staff were worried that patients who suffered from a significant co-morbidity could be at risk of not getting enough help for their psychiatric diagnosis if they chose to be treated with digital interventions.

*There isn’t much in-person contact with a therapist that way. And I think that if you have significant psychiatric comorbidity*,* a lot can be missed precisely from the psychiatric aspect if you don’t have this contact with a physical person. So that it is important to follow the patient’s condition*,* [for example]… if there is increased suicidality… [or]a patient who is perhaps about to enter a psychotic state*,* there are so many aspects that I think you can miss. (Informant 10*,* referring staff)*

### Theme 3: smoothly interlocking organizational gears

This theme illuminated the need for structured and well-functioning cooperation between the clinical outpatient settings at the SCDD and the ESU, in order to offer flexible treatment that includes digital content and in-person encounters, available to patients in an effective way.

#### Sub-theme 3a: A clear structure

Cooperation between the regular outpatient clinics and ESU was seen as fundamental to being able to further develop ESU. Another prerequisite for cooperation with the regular outpatient clinics to work was to have the management’s support for it as well as having the right tools, work methods, and processes in place to successfully achieve sustainable and effective cooperations. The informants described that a clear structure made it easier to follow the ESU guidelines for referring patients and made it clear how to share information necessary for the patients care, for example via their joint electronic medical record.

*I think the reason*,* if I may guess*,* that it [the patient influx] increases so much is that we have had time to think or to work out how our process should look….Awareness of [our ESU unit] is increasing all the time and we have had a very easy way to register with us….We wouldn’t have been able to do this if we hadn’t had management’s approval that we could kind of have the channels open and then just send all the patients who weren’t suitable for internet treatment into the clinic. So it has sort of been a prerequisite that all addiction clinics have taken the patients that they have the capacity for. There has…been that will [to make it work]*,* so I think it is very much about what decisions have been made at the management level. (Informant 5*,* ESU staff)*

The informants working at the ESU described that their work requires clear structure and framework to accommodate the growing number of patients and expanded staff since 2018. They described several goals, for example: to streamline care, reach more patients, and provide treatment efficiently. The informants perceived that digital systems could help improve patient care and reduce staff workload. The ESU staff and management described their focus on creating an effective system to manage the increasing patient load and ensure quality care.

*The number of patients has increased… and that means that we need this structure to handle that number…that we sort of know what we are doing in all steps and so on. So there will be a lot of work in the entire working group around those things.(Informant 9*,* ESU staff)*

At the same time, the ESU informants could see that through developing clear routines and guidelines, they have been able to create the clinical platform for digital interventions that they have today, meaning that they can continue to push forward towards new goals. The clear structure according to which the ESU works was seen as one of the major reasons for the success of ESU as a clinic. For the ESU´s work to be successful, it was experienced as important to have well-organized processes for defining work tasks, prioritize work as well as communication, and for following up on work to establish new areas of digital treatments.

*It is very structured. It has a very clear entry routine. It is also easy because it is very clear which interventions we offer*,* so it is also quite easy to assess the patient*,* i.e. if you look at the difference from a local addiction clinic*,* it is quite easy to see that this patient should be with us*,* these patients should not be. It is quite easy to create structured care flows because [what we do] is based on applying for a program and then we assess whether the patient should attend that program… (Informant 5*,* ESU staff)*

#### Sub-theme 3b: Cooperation and communication

Several informants emphasized that cooperation between the ESU and their outpatient clinic functioned well, facilitated by a shared electronic medical record (EMR) system. The EMR system was perceived as an effective and structured tool that streamlined patient management, improved continuity of care, and enhanced communication between units. The ability to access real-time patient information when the patient had simultaneous contact with ESU and regular outpatient care, allowed healthcare providers to monitor progress, identify when additional support was needed, and ensure that patients received appropriate care. However, informants also highlighted the importance of communication beyond the EMR, particularly in urgent situations where immediate action was required. Instances such as patient relapse or suicidal thoughts or plans necessitated direct and timely communication to coordinate care interventions swiftly. While the EMR provided a solid foundation for cooperation, informants valued the ability to connect with colleagues through other channels when urgent matters arose.

Additionally, informants appreciated that cooperation between the ESU and their outpatient clinic remained effective year-round, including during holiday periods such as spring, summer, and winter breaks. This continuous availability ensured that referrals to the ESU could be processed without delay and that patients could receive timely care when needed. The joint EMR was seen as not only facilitating coordination between healthcare providers but also strengthening the overall quality of care by allowing for proactive interventions.

*I think it works very well*,* and we can always see if the patient is not properly supported. We also follow… [the patient because] we have an open medical record between us. We can easily see when the patient is not doing what they are supposed to… I think it is easy to cooperate with a common medical record.* (Informant 4, referring staff)

While several informants experienced good cooperation with the ESU, they also expressed a desire to further develop their communication. For the most part, the referral process functioned well, but staff wanted to establish a stronger connection between referring units and the ESU. Some informants described a sense of uncertainty when referring patients, likening it to sending them “into outer space.” This feeling seemed to stem primarily from the fact that digital treatment was still relatively new and did not yet feel as familiar or intuitive as referring patients to conventional care. They expressed a need for greater insight into what happens to patients after a referral is made to the ESU, helping them feel more confident in the cooperation process.

To bridge this gap, referrers suggested implementing a secure communication platform for internal chat-based communication that would shield patients’ sensitive personal information, to facilitate digital meetings and discussions while maintaining patient confidentiality. They believed that such a tool could foster a sense of connection, allowing for more direct interaction with ESU staff and enhancing cooperation. By strengthening these ties, healthcare providers could feel more engaged in the digital treatment process, ultimately improving patient outcomes and ensuring smoother transitions between services.

*Most of the time we have some collaboration with those we collaborate with*,* if we were to have some type*,* maybe a chat or something*,* where we see those who work*,* maybe talk a little more about patient matters. See who it is we collaborate with*,* because it feels like they sit there*,* we sit here and we don’t really know. We don’t really know who they are*,* so it would probably be good around these patients.* (Informant 4, referring staff)

In this study, the findings indicate that all informants held a positive view of digital interventions, recognizing them as a valuable complement to existing treatment options. Staff members highlighted that referring patients to digital interventions alleviated some of their workload. However, perspectives varied regarding the optimal implementation and integration of these interventions within the healthcare system. A key point raised by one informant was whether digital interventions should remain within a separate unit or be integrated into the regular workflow of outpatient clinics. This informant suggested that incorporating digital interventions into standard clinical practice could enhance accessibility and flexibility for both patients and staff, ultimately expanding the reach of care. Such integration was seen as a way to streamline treatment delivery and improve patient access while optimizing clinical resources.

*Now it’s a separate unit and maybe there are advantages to that*,* but I think it’s actually desirable to… to increase collaboration and to make care a little more accessible and a little more flexible and actually reach more people. (Informant 3*,* referring staff)*

#### Sub-theme 3c: optimizing available resources

Using digital interventions can facilitate redistribution of resources, so that they are used in the best possible way. Resource optimization in healthcare involves efficiently utilizing available resources such as staff, equipment, and time to deliver high-quality care to as many patients as possible This, in turn, means that patients who need more extensive interventions can receive them, while patients who need less extensive interventions can get help via digital interventions, at least as a start. By offering digital interventions and more advanced on-site care when needed, resources can be used effectively to ensure that every patient receives the appropriate treatment. This approach not only improves care but also reduces costs and allows for more expensive care to be provided to those who need it. Ultimately, it aims to tailor care to individual needs and optimize resource utilization in healthcare settings.

*Being able to differentiate care*,* to offer simpler care to those who need simpler care and then more advanced care on site when they need it so that it’s really about resource optimization to use our resources in the best way and that everyone should get the treatment that suits them just right. (Informant 2*,* manager)*

It is crucial to respond to current patient demands with available resources while also preparing for an anticipated increase in future patient needs. Several of the informants with managerial backgrounds envisioned a future where the demand for healthcare would surpass current levels, requiring proactive planning. Limited resources currently hinder the capacity to meet existing patient needs, necessitating the development of digital healthcare to address future demands effectively. The focus is on expanding opportunities for patients to receive necessary care through self-monitoring, with the understanding that not all patients will be able to manage this independently. The implementation of hybrid variations of care, such as blended care, is seen as essential, but would require a more intuitive digital system than what is currently available. The informants emphasize the potential of digital interventions to efficiently reach more patients at a lower cost, paving the way for a new, smarter approach to healthcare delivery.

*Today*,* we have contact with 1% of the total population in the county in dependency care… I usually say that maybe we should be in contact with…maybe even 3%… And we will never be able to…triple our budget to take care of these patients physically… we have to think in a new… smart way and then digital contact care contacts are the option that feels most possible and where we can reduce the unit costs quite significantly and reach a lot more people who maybe today shy away from going to an in-person clinic. (Informant 12*,* manager)*

## Discussion

The study explored the benefits of digital interventions for patients with alcohol use disorders in specialized addiction care, identifying three key themes. *An easy way in* suggested that digital interventions were needed in order to expand low-threshold accessibility, thus allowing more patients to receive flexible and individually tailored care. *Meeting individual patient needs* emphasized the importance of delivering treatment at the right time and in accordance with each patient’s specific circumstances. *Smoothly interlocking organizational gears* highlighted the necessity of clear structures, transparent guidelines, and cooperation between clinics to optimize resources and ensure that patients receive appropriate support. The informants in the study envisioned that offering digital interventions as a complement to routine care could not only enable the treatment of more patients simultaneously in a cost-effective manner but also help reduce healthcare queues and improve patient outcomes.

A particularly positive component identified in this study was the presence of a clear workflow, which contributed to a well-functioning cooperation with the ESU. This finding aligns with previous research indicating that a well-defined workflow facilitates the implementation of digital interventions [34]. Additionally, prior studies emphasize the importance of establishing methods for cooperation between units both before and during implementation to ensure successful integration of digital tools into clinical practice [35].

In the present study, informants viewed the option of referring patients to the ESU as highly beneficial, as it provides an additional treatment option that eases their workload while ensuring patients access to digital interventions. The implementation of digital interventions within the ESU has progressed due to the presence of clear referral guidelines, which have helped streamline the process and enhance its efficiency. However, for the continued successful implementation of digital interventions, it is essential to evaluate both the feasibility and efficiency of existing workflows to ensure sustainable integration into addiction care [34]. Furthermore, ongoing refinement of communication and cooperation strategies between units may further enhance the effectiveness of digital interventions, ensuring that they serve as a truly complementary and well-integrated components of specialized addiction care.

The role of theoretical frameworks in implementation science and psychological treatment has been extensively discussed in recent literature. In contrast, another study took a broader perspective by addressing challenges in the effectiveness and dissemination of psychological treatments. While their focus was not on theory selection per se, they underscored the necessity of refining theoretical models for psychological disorders and enhancing implementation strategies to improve patient outcomes [36]. Taken together, these studies highlight the need for a more deliberate and systematic application of theory in both implementation science and clinical practice [36–38].

**Fig. 2:**
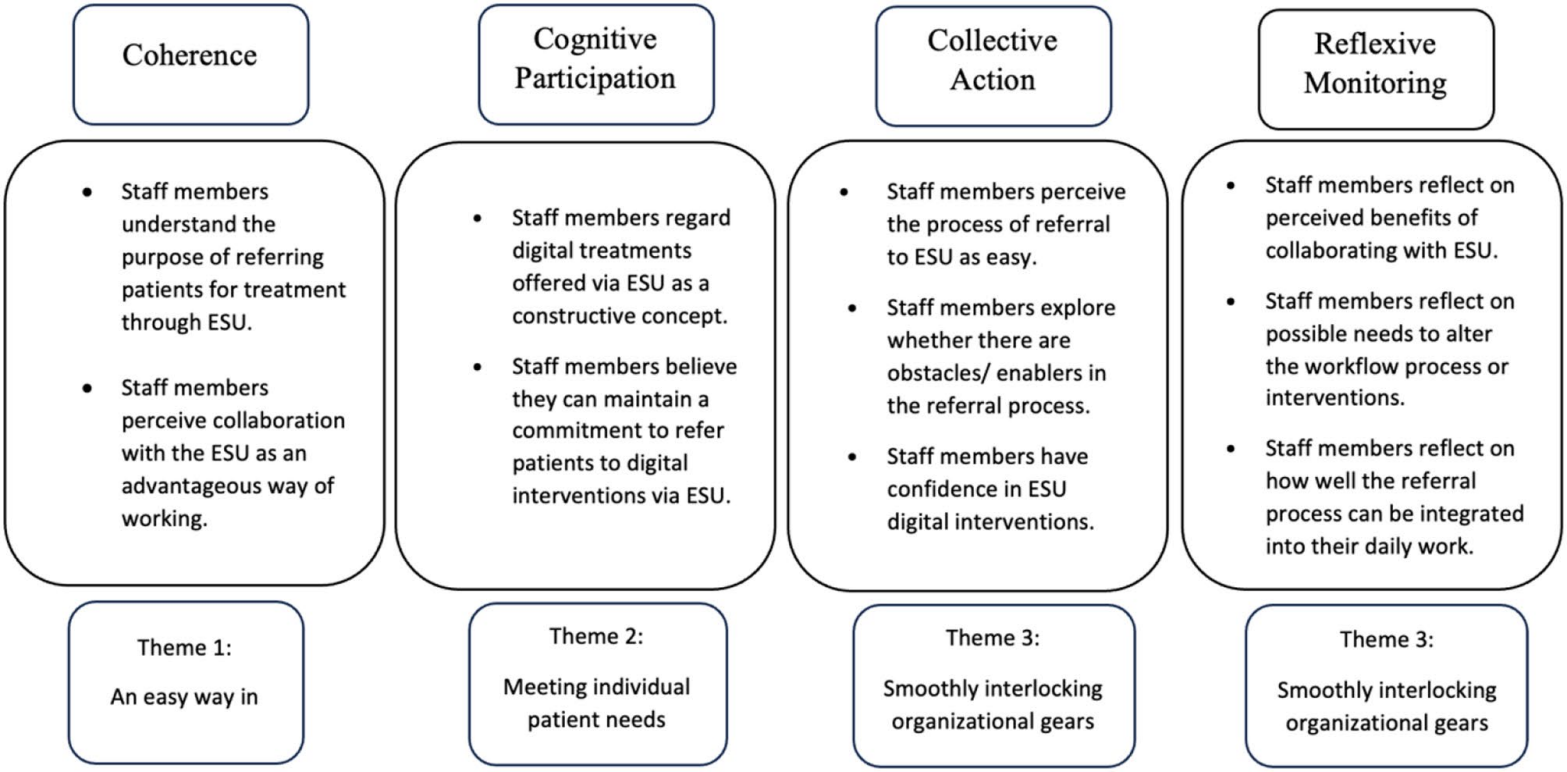
Normalization process theory as a context for understanding study results

We therefore now turn to a discussion of the themes and sub-themes that emerged in the present study in relation to NPT, conceptualized in the overview of interrelated NPT concepts and study themes shown in Fig. 2. We have chosen to use NPT to increase our understanding as a framework that clarifies processes involved in implementing new technologies and interventions, as well as evaluation of such processes. NPT also provides a visualization of how these processes can be integrated and maintained in daily work [39]. Also, NPT theory can help explain how the implementation of digital interventions described in this study can be successfully optimized through adaptation to the healthcare staff’s competence and goals [40].

Based on a general interpretation of NPT, its first construct, *Coherence*, can be described as the sense-making work people do in relation to a new practice; it includes the components of *differentiation*,* communal specification*,* individual specification*, and *internalization* [39]. In this study, informants expressed positive perceptions of digital interventions, highlighting the benefits of expanding patient support. The informants emphasized the importance of staff understanding these interventions to effectively refer patients. This is also highlighted as important according to the Coherence construct, where the internalization component shows how sense-making as important as it leads to understanding– and internalizing - the value and importance of a certain practice [16]. Previous studies have shown that a lack of understanding or uncertainty among staff about the benefits of digital interventions in psychiatric and addiction care were viewed as obstacles for implementation [12, 13]. In this study, the Coherence construct [16] indicates that it is important that the staff understand the purpose of offering digital treatment for their patients. The informants describe their internalization of purpose when they state that implementation of digital interventions can reduce the stigmatization of the patient group, contributing to more people seeking care as healthcare offerings become more flexible. This evidence of internalization indicates progress towards successful implementation of digital interventions in the specific addiction setting investigated.

The construct of *Cognitive participation* involves the relational work that staff need to do, to establish and maintain a community of practice for implementing new working methods. This includes activities like *initiation*,* enrollment*,* legitimation*, and *activation*, which help foster a sense of belonging among staff and engage them in the process of implementing digital interventions [39]. The success of implementing digital interventions relies on the engagement of users, including staff and patients, in real environments. Understanding factors that affect engagement can lead to development of strategies to overcome barriers to implementing these interventions effectively. Involving staff in the development and design of interventions could increase their feeling of participation in the continued integration of digital interventions into routine addiction care and lead to a greater likelihood of successful integration into routine addiction care. Recent research in primary care has suggested that effective implementation of digital interventions for alcohol use disorders (AUD) could increase treatment options for addiction in the primary care setting if strategies such as clinician training and dedicated clinician time were introduced [41]. Such engagement activities can lead to an increased sense of participation that results in improved flexibility in providing in-person and digital interventions, as well as better adaptation of treatment to individual patient needs. Such activities could also facilitate staff attunement to patient opinions on their needs in relation to digital interventions, as well as to how blended care formats could best be further developed. Tailoring the introduction of digital interventions for AUD in primary care to the needs of patients with varying AUD severity has been seen as crucial [41]. In relation to the Cognitive participation construct, the informants in the present study further highlighted the importance of offering multiple ways to receive and exit care, such as starting with internet-based treatment and transitioning to in-person support if needed. There should be flexibility to move care between physical and digital contexts to ensure continuity of treatment.

The construct of *Collective action* focuses on how healthcare staff engage in digital interventions. It involves *interactional workability*,* relational integration*,* skill set workability*, and *contextual integration* for successful implementation [39]. Clear communication and cooperation are essential for operationalizing a new method [42]. Identifying staff skill sets is crucial for determining new roles needed for implementing digital interventions. In the present study, concerns about increased workload were raised, but clear and structured work routines were seen as alleviating confusion about task assignments. However, developing effective communication strategies and structured patient referrals is also vital for successful digital intervention adoption, as shown in previous studies [43, 44]. Insufficient digital skills can lead to reluctance among healthcare professionals to use digital solutions; it is therefore important for organizations to regularly allocate resources for employee training [45]. In the context of NPT, *skill set workability* is essential for successful digital intervention implementation [16]. Informants found the ESU interventions easy to use, with clear referral guidelines. While minimal training was needed, they expressed interest in learning more about the interventions and their patient suitability, demonstrating commitment to integration.

Our study highlighted the importance of structured routines and clear guidelines for effective use of digital tools. Informants indicated that existing workflows supported the integration of digital interventions, while expressing a need for more training, suggesting that skill development is crucial for sustaining these innovations. In line with NPT, successful integration depends on both technical feasibility and staff’s ability to adapt practices to their skills [16]. Ongoing training and support are essential to optimize staff skills for digital interventions, especially as the demand for flexible care increases. Ensuring healthcare providers have the necessary skills will be key to the long-term sustainability of digital healthcare [16]. Thus, addressing skill set workability within the NPT framework helps inform strategies for overcoming challenges in the widespread implementation of digital interventions across healthcare systems [16]. In addition to skill development, engagement plays a critical role in the success of these interventions. A recent study highlighted the transformative potential of digital solutions in psychiatric treatment, emphasizing the importance of engagement at multiple levels and collaboration across disciplines. Addressing engagement as an implementation issue is essential for maximizing the clinical impact of digital interventions on mental health care [46].

The final construct, *Reflexive monitoring*, is a crucial aspect of assessing the impact of new digital interventions on participants and their effectiveness. It involves systematically collecting information on the utility and progress of the interventions to identify areas for improvement. This process includes *systematization*,* communal and individual appraisal*, and *reconfiguration* of interventions [39]. The present study highlights the importance of digital tools being clear, easy to implement, and beneficial for healthcare staff. While digital interventions offer these benefits in the form of efficiency, flexibility, and cost-effectiveness, it is essential for managers and staff to evaluate the impact of digital intervention implementation on their work and make necessary adjustments to ensure the intervention is easier to use in their daily work. Ultimately, the success of digital interventions relies on staff feeling that these interventions are feasible to work with and that they contribute to improving patient care as well as digital interventions being easy to integrate in their daily work. In the present study the informants described how digital interventions could contribute to more patients receiving the care they need, with a faster start to treatment. To ensure that digital treatment programs function as expected, it is important to continue to evaluate the interventions from the perspectives of referring staff, patients, as well as treatment providers. In this regard, Murray et al. (2010) suggest that NPT theory provides a reliable framework for evaluating and enhancing complex interventions, and addresses the problem of research not being translating into practice. Utilizing NPT can improve intervention development, contribute to trial effectiveness and facilitate introduction into the healthcare system [47].

Facilitating factors for implementation according to NPT are useful when considering how to improve implementation digital interventions within addiction care. One key factor is staff understanding the purpose and value of digital interventions (Coherence). When staff internalize these benefits—such as reducing stigma and increasing access to care—their motivation to recommend them increases. This aligns with studies emphasizing that sense-making is crucial for successful implementation [12, 13]. Additionally, staff engagement (Cognitive Participation) is greater when they are involved in developing and adapting digital interventions. This supports research highlighting the importance of participation in change processes to enhance acceptance [41]. Nonetheless, some gaps remain regarding the usefulness of NPT to achieve sustainable implementation. One such gap concerns staff’s digital skills and their impact on using digital tools (Collective Action). Informants expressed a need for structured training and support, aligning with research showing that limited digital competence can hinder implementation [45]. Another gap concerns the need for ongoing evaluation and adjustment of interventions (Reflexive Monitoring). While informants recognized the benefits of digital treatment, they stressed the importance of continuously assessing patient experiences and treatment effectiveness, supporting previous findings on the necessity of structured follow-up mechanisms [47].

In sum, our findings align with previous research emphasizing the importance of clear communication, structured routines, and staff involvement in facilitating implementation [41, 43, 44]. However, the study also highlights certain limitations within the NPT framework, particularly its limited consideration of patient expectations and experiences—factors that significantly influence the acceptance and sustained use of digital interventions. While NPT effectively structures implementation analysis by focusing on professional engagement, it does not fully account for the critical role of patient perspectives. Furthermore, the framework overlooks key technological challenges, including usability, interoperability, cybersecurity, and data privacy, all of which are essential for the successful adoption and normalization of digital health tools. To develop a more comprehensive understanding of digital health implementation, NPT should be complemented with models that explicitly incorporate patient engagement and technological feasibility. Future research would benefit from integrating NPT with other theoretical frameworks to provide a more nuanced perspective on the complex interplay between organizational, technological, and patient-related factors in digital health adoption.

### Strengths and limitations

The current study was conducted within a specialist addiction care setting, providing a focused exploration of digital interventions in this context. A key strength of the study was that all informants were recruited from units that actively refer patients to the ESU, meaning they had firsthand experience with the service. This allowed them to provide nuanced, experience-based insights into both the benefits and challenges of digital interventions. Furthermore, all informants were well-acquainted with addiction as a medical and psychological field and possessed a clear understanding of the available treatment options.

Another strength of the study was its inclusion of informants from different professional backgrounds, ensuring that the topic was examined from multiple perspectives. The study incorporated insights from practitioners working directly with digital interventions, referrers responsible for patient referrals to the ESU, and clinical managers overseeing the broader treatment framework. Integrating these perspectives has facilitated a comprehensive understanding of how digital interventions function within specialized addiction care, shedding light on both practical implementation and, to some extent, strategic decision-making processes.

Some limitations should also be noted. One limitation was that the study was carried out in a setting in the Stockholm metropolitan region. Even though the ESU treats patients from all over the country, it is not possible to fully generalize this study to Sweden as a whole, as the cooperation described took place only between the ESU, within the SCDD. Furthermore, given that the study was carried out at a specialized addiction care clinic in Stockholm, Sweden, there is a risk that the results cannot be applied in other countries with different healthcare systems. A third limitation concerns the question of data saturation, which occurs when no new themes or codes emerge from the data. In this study, EP interviewed all the informants, meaning that assessment of saturation was only carried out by one researcher, who perceived that much of the emerging information was repeated after a certain number of interviews. This can be defined as a limitation, although saturation can be viewed as a subjective experience, as discussed by Braun and Clark [48].

## Conclusions

Overall, this study identifies key factors influencing the successful integration of digital interventions in addiction care, based on insights from staff across three professional groups. Clear workflows, staff engagement, structured communication, and continuous evaluation by the ESU were highlighted as critical for effective implementation. Informants viewed digital interventions positively, particularly for their potential to reduce stigma and improve accessibility, while also emphasizing the need for structured training and flexible care pathways. These insights offer valuable guidance for integrating digital technology into existing intervention among staff accustomed to traditional in-person care.

Using NPT as a framework, our findings underscore the importance of staff understanding the purpose of digital interventions (Coherence), actively participating in their implementation (Cognitive Participation), integrating them into routine practice (Collective Action), and continuously assessing their impact (Reflexive Monitoring). To enhance understanding of digital health incorporation into healthcare, future research should complement NPT with models that account for patient engagement and technological feasibility, offering a more comprehensive perspective on the interplay between organizational, technological, and patient-related factors.

## Electronic supplementary material

Below is the link to the electronic supplementary material.

Supplementary Material 1

Supplementary Material 2

## Data Availability

No datasets were generated or analysed during the current study.

## Abbreviations

AUD: Alcohol use disorder
EMR: Electronic Medical Record
ESU: e-Support Unit
NPT: Normalization process theory
SCDD: Stockholm Centre for Dependency Disorders
WHO: World Health Organization
