# Supplementary material for: Digital interventions as part of routine addiction care in Sweden: healthcare staff perceptions of what works
Source: Addict Sci Clin Pract. 2025 May 12;20:41. doi: 10.1186/s13722-025-00570-1 (PMC12067725; doi:10.1186/s13722-025-00570-1)
Supplement: Supplementary file 1 — Supplementary Material 1 [file 13722_2025_570_MOESM1_ESM.docx]

**Additional file 1. Interview guide**

**For healthcare staff at the e-support unit (ESU):**

1. Could you tell me how the ESU was created?

2. How has the ESU developed since it started (or since you started working there)?

3. Could you briefly describe your work at the ESU?

4. Please describe what you think works well in terms of the patient flow at the ESU? (How has it varied over time?)

5. Please explain the referral procedure for ESU?

6. Does anything need to be improved in terms of patient flow? If so, please describe.

7. How do you see your continued work at ESU?

8. What are your thought about treating patients with co-morbidity via the internet? Can you see any obstacles? Benefits? Something that would need to change?

9. Do you feel that the patient’s co-morbidity affects care from the ESU? If so, how?

10. Is there anything you would like to add before we finish this interview?

**For staff at units within the Stockholm Center for Dependency Disorders (SCDD) referring patients to the ESU:**

1. Please briefly describe your unit and your role there.

2. Please describe your unit's patient group.

3. What do you know about the ESU?

4. What makes you choose to refer a patient to ESU?

5. How does the referral procedure to the ESU work? (Possible follow-up question 1: How does it work for patients and staff respectively? Possible follow-up question 2: Please describe what works well and what could be improved.)

6. Do you feel that there are any advantages and disadvantages to treating patients with co-morbidities via the Internet? Is there anything that would need to change?

7. How do you see your collaboration with ESU developing in the future?

8. Is there anything you would like to add before we finish this interview?

**For clinical managers working at Stockholm Center for Dependency Disorders (SCDD):**

1. Please briefly describe your role at the SCDD.

2. Please describe the SCDD patient group in general.

3. What do you know about the ESU?

4. What patients do you think should be referred to the ESU?

5. What do you know about the referral procedure to the ESU? (Possible follow-up question 1: What do you know about how it works for patients and staff respectively? Possible follow-up question 2: What do you know about what works well and what could be improved in the referral procedure and collaboration with the ESU?)

6. What advantages and disadvantages do you perceive to treating patients with co-morbidities via the Internet? Is there anything that would need to change?

7. How do you see collaboration with the ESU - within the SCDD and outside it - developing in the future?

8. Is there anything you would like to add before we finish this interview?
